# Delivery of genetic medicines for muscular dystrophies

**DOI:** 10.1016/j.xcrm.2024.101885

**Published:** 2025-01-06

**Authors:** Yulia Chulanova, Dor Breier, Dan Peer

**Affiliations:** 1 Laboratory of Precision Nanomedicine, The Shmunis School of Biomedicine and Cancer Research, George S. Wise Faculty of Life Sciences, Tel Aviv University, Tel-Aviv, Israel; 2 Department of Materials Sciences and Engineering, Iby and Aladar Fleischman Faculty of Engineering, Tel Aviv University, Tel Aviv, Israel; 3 Center for Nanoscience and Nanotechnology, Tel Aviv University, Tel Aviv, Israel; 4 Cancer Biology Research Center, Tel Aviv University, Tel Aviv, Israel

**Keywords:** muscular dystrophy, gene delivery, gene therapy, gene editing, adeno-associated virus, lipid nanoparticle, polymer nanoparticle, extracellular vesicles, genetic medicines

## Abstract

Muscular dystrophies are a group of heterogenic disorders characterized by progressive muscle weakness, the most common of them being Duchenne muscular dystrophy (DMD). Muscular dystrophies are caused by mutations in over 50 distinct genes, and many of them are caused by different genetic mechanisms. Currently, none of these diseases have a cure. However, in recent years, significant progress has been made to correct the underlying genetic cause. The clinical development of adeno-associated viral vector-based therapies has simultaneously produced excitement and disappointment in the research community due to the moderate effect, making it clear that new methods of muscle delivery have to be created. Herein, we review the main characteristics of major muscular dystrophies and outline various muscle-targeted delivery methods being explored for genetic medicines.

## Graphical abstract

**Figure:**
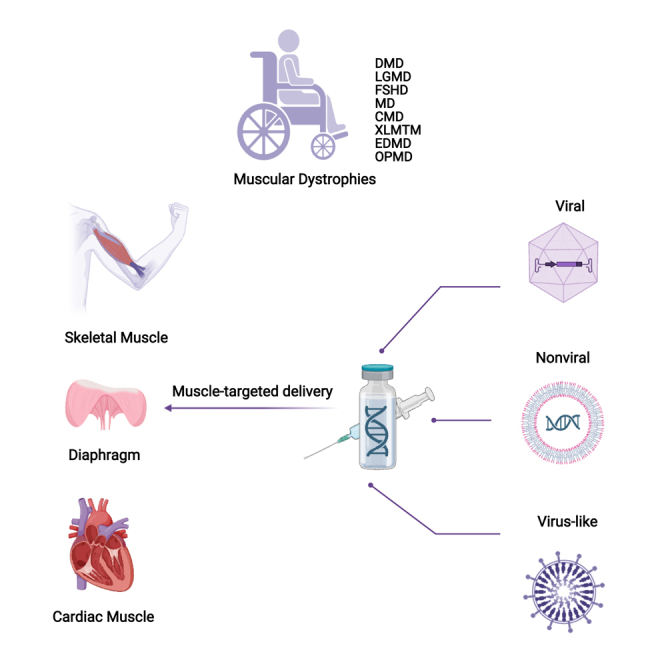

## Teaser

Effective genetic medicines for muscular dystrophies require efficient delivery methods. While some approaches, mostly viral, already exist, there is room for improvement. Chulanova et al. describe both well-established and emerging methods of gene delivery to muscles and stress the need for tailoring to a specific muscular dystrophy type.

## Introduction

Muscular dystrophies (MDs) comprise a heterogenic group of disorders that are characterized by progressive muscle weakness and wasting. Different forms of MD can exhibit differences in severity, age of onset, and life expectancy and may affect various muscle groups differently. Certain MDs involve not only skeletal muscles but also present cardiomyopathy features.^1^

Aside from Duchenne MD (DMD), none of these diseases currently have any treatment, with the standard of care consisting of symptom management. However, there is a constantly growing amount of research aimed at developing genetic medicines to cure MD. Currently, most of the research is focused on DMD due to its well-known disease mechanism and relatively high abundance in the population. Table 1 summarizes genetic causes and affected muscle groups in an attempt to highlight the differences of the various phenotypes, which could serve as selection criteria for a specific delivery vehicle. More detailed information about mutations and clinical manifestations of every kind of MD has been summarized elsewhere.^1^

**Table 1:** Characteristics of the main muscular dystrophy types

Muscular dystrophy	Gene	cDNA length	Mutation type	Muscles affected	Prevalence
Duchenne muscular dystrophy	DMD	14 kb	loss of function	skeletal muscles, heart, and diaphragm	4.78/100,000 males^2^
Becker muscular dystrophy (BMD)	DMD	14 kb	loss of function	hips, pelvis, thighs, shoulders, and calves	1.53/100,000 males^2^
Limb-girdle muscular dystrophy (LGMD)	39 genes (see Bouchard et al.)	1 kb–101 kb	loss of function	upper arms, upper legs, hips, and shoulders	1/14,500–1/123,000^3^
Facioscapulohumeral muscular dystrophy type 1 (FSHD1)	DUX4 (repeat contraction)	N/A	epigenetic de-repression	face, shoulder girdle, and upper arms	total FSHD: 5–12/100,000^4^ FSHD2: about 5% of total FSHD population
Facioscapulohumeral muscular dystrophy type 2 (FSHD2)	SMCHD1, DNMT3B, and LRIF1	6 kb, 2.5 kb, 2.3 kb	loss of function		
Myotonic dystrophy (DM)	DMPK CNBP	N/A	repeat expansion	distal, face and neck, heart, and diaphragm	1/8,000^5^
Congenital muscular dystrophy (CMD)	35 genes (see Zambon et al.)^6^	1 kb–10 kb	loss of function	skeletal muscles, heart, and diaphragm	0.99/100,000^7^
X-linked myotubular myopathy (XLMTM)	*MTM1*^6^	1.8 kb	loss of function	skeletal muscle	2/50,000 males^8^
Emery-Dreifuss muscular dystrophy (EDMD)	10 genes, most common—LMNA and EMD (see Heller et al.)^4^	1 kb–101 kb	loss of function	calves and biceps and heart	0.39/100,000^7^
Oculopharyngeal muscular dystrophy (OPMD)	PABPN1	N/A	repeat expansion	Muller’s muscle, proximal limbs, and cricopharyngeus muscle^9^	1:100,000–1:1,000,000^9^

As of today, there are several therapeutic approaches: gene replacement, gene silencing, and gene editing. Due to the heterogeneous nature of MDs, the therapeutic approach must be tailored to the specific type of mutation. The underlying mutation causing the disease could be loss-of-function mutations, epigenetic de-repression causing unwanted protein activity, and repeat expansion also leading to insufficient protein expression.^1^

**Figure 1:**
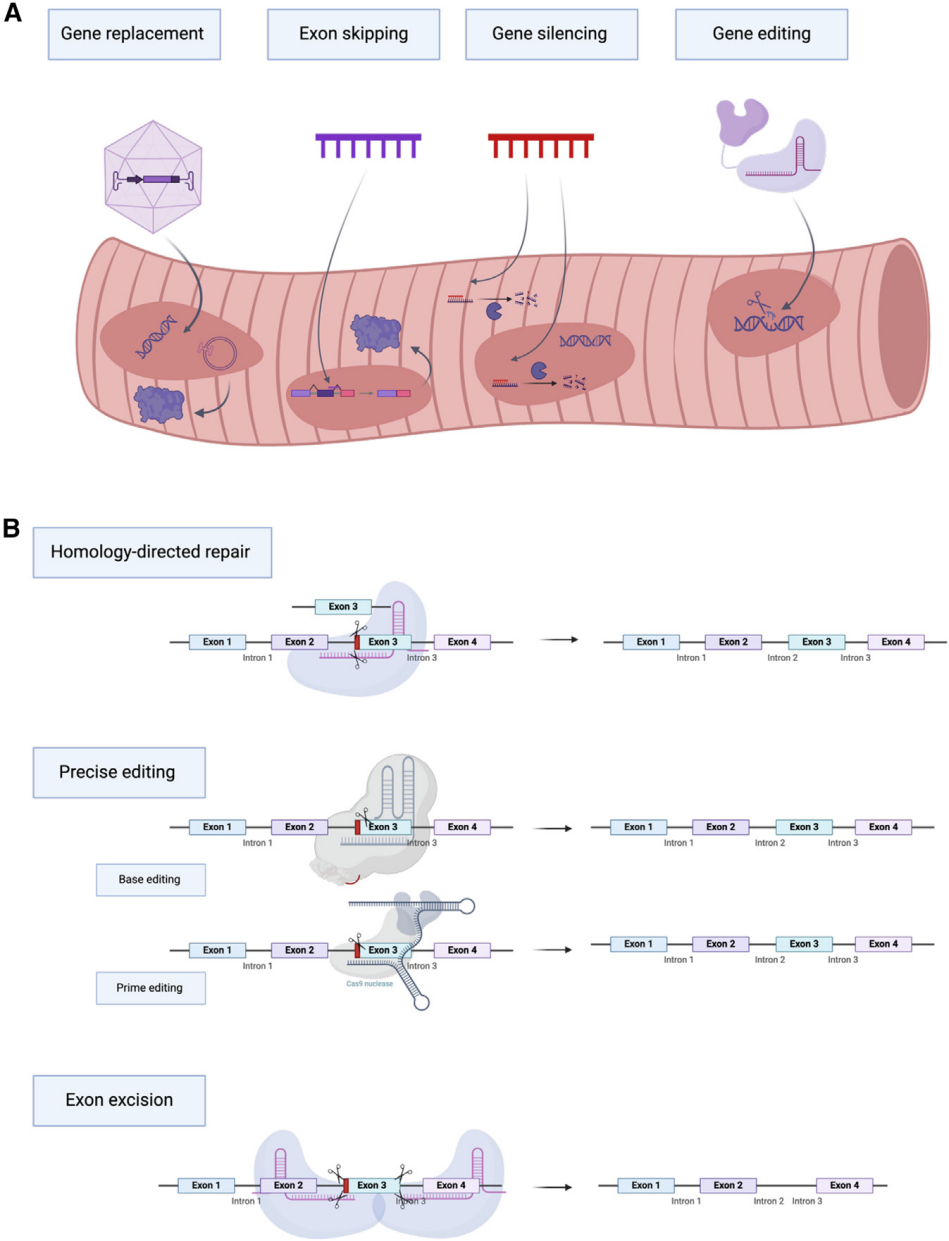
Gene therapy methods for muscular dystrophy treatment (A) General methods applicable to various cases of muscular dystrophies. (B) Gene editing methods under development. A hypothetical patient carries a mutation in exon 3 (in red). Homology-directed repair is a classical method of gene correction using a donor DNA template, which replaces the target region upon a double-stranded break (DSB), followed by homology-directed repair via cellular DSB repair mechanisms. Precise editing methods are capable of correcting specific point mutations or small deletions and insertions and include but are not limited to base editing and prime editing. Exon excision methods aim to cut out one or several exons to remove mutations in a specific region, producing an altered but still functional protein.

Gene replacement therapies are applicable to loss-of-function mutations and aim at substituting the broken gene with its functional copy by delivering nucleic acids into the cells (Figure 1A). A major obstacle in this sort of therapy is the need for the long-term (ideally life-long) persistence of the delivered genetic material, a feature that was only demonstrated when using viral vectors.^10^ However, viral delivery is currently hindered by the lack of technologies for muscle-specific delivery of large genes, such as dystrophin (for DMD), whose coding sequence exceeds 13 kb^11^

For mutations leading to the production of a toxic protein, a gene-silencing approach can be used (Figure 1A). Gene silencing is mostly based on the RNA interference mechanism, which is successfully used in clinical practice for several other diseases, such as hereditary transthyretin-mediated amyloidosis,^12^ acute hepatic porphyria,^13^ and lowering low-density lipoprotein levels with inclisiran.^14^ However, as gene silencing is rarely applicable to MDs, it is not explicitly discussed in this review.

Another approach involves gene editing (Figure 1A). With the discovery of CRISPR-Cas9 system, gene editing technologies took the spotlight.^15^ The CRISPR-Cas9 system is capable of cutting the double-stranded DNA in a specific location, therefore allowing to knock out the malfunctioning gene or knock in the correct sequence when using a DNA template.^15^ However, the efficiency of the latter is still too low for therapeutic applications, leading many researchers to opt for more creative technologies. For example, the excision of several exons can help restore the reading frame and achieve the production of a shortened but functional protein, thus converting the disease to a milder phenotype (Figure 1B).^16^ The other option is using more recently developed gene editing technologies such as base and prime editing to directly repair the mutation far more efficiently than the traditional knockin system. This approach, however, requires separate testing for each mutation, which is a challenge considering the tremendous variety of mutations for each phenotype. Notably, although CRISPR-Cas9 still requires the delivery of long genetic sequences, the field is rapidly developing with the discovery of new, smaller gene editing systems.

A special place among genetic therapies belongs to RNA-modifying drugs, specifically exon-skipping antisense oligonucleotides (ASOs).^17^^,^^18^ Exon-skipping ASOs act by binding to the pre-mRNA and interfering with the inclusion of the exon neighboring a mutated exon to restore the reading frame (Figure 1A). Several ASO drugs for MDs have been approved to date, namely eteplirsen, golodirsen, viltolarsen, and casimersen. Despite the approval by the Food and Drug Administration (FDA), these drugs are subject to controversy. First, the levels of *de novo* dystrophin production were low (e.g., 0.9% after 3.5 years for eteplirsen); second, *de novo* dystrophin is not fully functional due to the lack of an exon; third, approval was based on surrogate biomarkers that do not perfectly represent the patient’s condition.^17^^,^^18^ The low level of *de novo* dystrophin is thought to result from the low delivery efficiency to muscle nuclei via intravenous injection, an inherent problem associated with non-ionic backbones of phosphorodiamidate morpholino oligonucleotides.^19^

Herein, we focus on currently used and emerging technologies for both viral and non-viral systemic muscle delivery of genetic material. In addition, to highlight the differences in delivery vehicles applicable to each case, we describe the characteristics of all major types of MDs, including the type of mutation and muscle groups affected to appreciate the difference in delivery vehicles applicable in each case. Finally, we point out structural characteristics of muscle tissue that are likely to play a role in the selection of an ideal delivery vehicle.

## Muscle structure

To appreciate gene therapy development hurdles, it is essential to understand the structural features of the muscle. In this review, we briefly discuss the features that appear important when selecting a payload-vehicle pair for genetic medicine development.

Muscle tissue is divided into three types: skeletal, cardiac, and smooth, with each performing specific functions. Here, we discuss skeletal and cardiac muscle structure due to their involvement in MD pathology.

**Figure 2:**
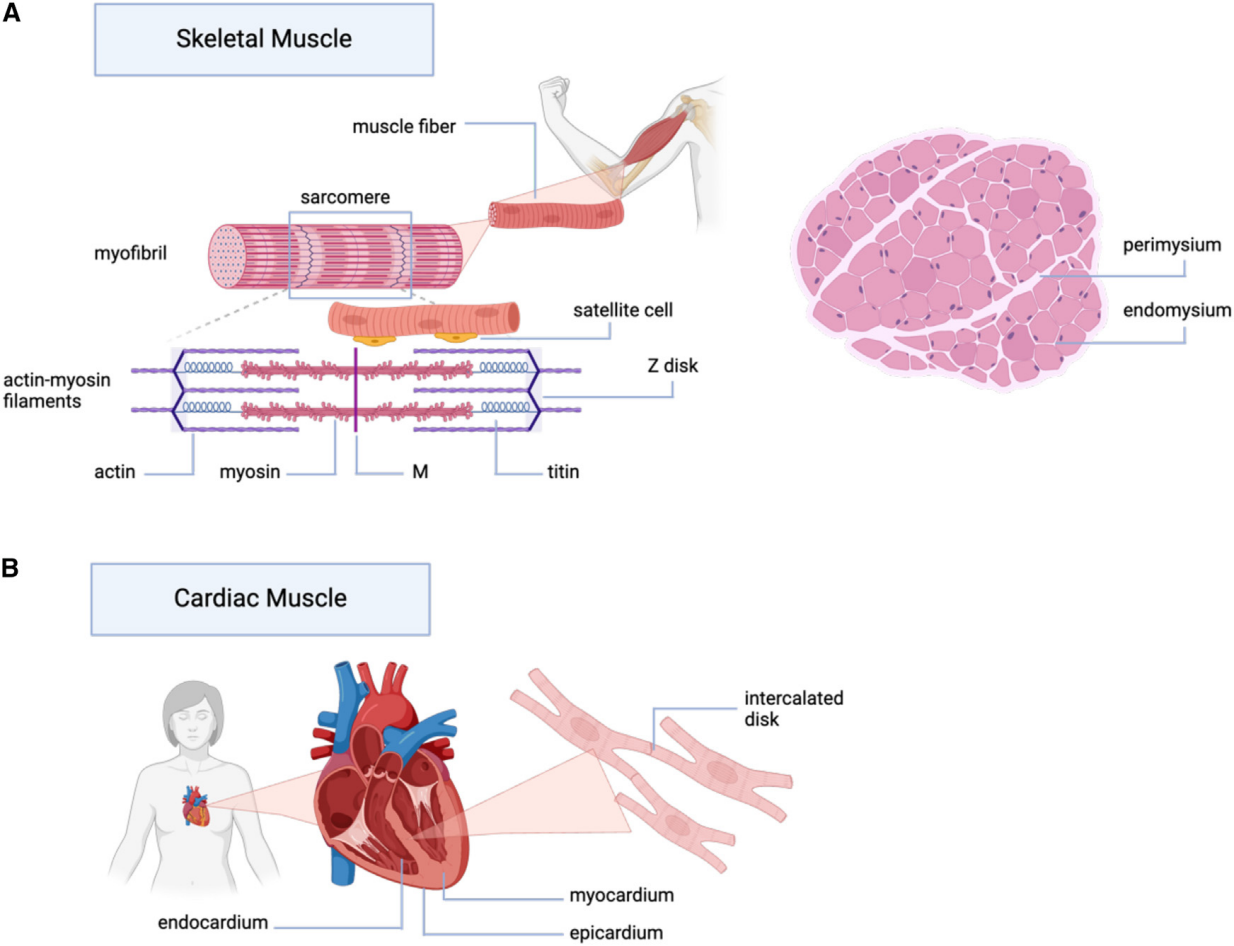
Schematic illustration of muscle structure (A) Skeletal muscle. Muscle fibers are composed of myofibrils, which are in turn composed of actin and myosin filaments. Muscle fibers are packed together to form muscle fascicles. Satellite cells responsible for muscle fiber restoration are located on the side of the multinucleated muscle fiber. On the right, a healthy skeletal muscle cross-section, showing connective tissue layers (perimysium and endomysium) and peripherally positioned nuclei. (B) Cardiac muscle. Muscle cells are found in the myocardium, between the epicardium and the endocardium. The myocytes are branched and connected through intercalated discs. There are no specific cells that are responsible for the renewal of the myocardium.

Skeletal muscles comprise about 30% of body weight; they are essential for any body movement and to maintain posture and position. The main contractile unit of the muscle is the sarcomere, which consists of multinucleated muscle fibers held together with connective tissue (Figure 2A). The muscle fibers are made up of myofibrils, and each myofibril is itself a set of myofilaments. The two main proteins taking part in contraction are actin and myosin, which are arranged into thin and thick filaments, respectively, and follow a striated pattern. As these muscle fibers are terminally differentiated and cannot divide, any damaged muscle tissue is repaired with the help of satellite cells. The non-dividing state of the multinucleated muscle fiber is important when selecting a delivery vehicle since delivery of a non-integrating payload can be expected to persist in those fibers over a longer period. Satellite stem cells (SCs) are located between the basal lamina and the sarcolemma and serve as the only way to replenish the damaged fiber.^20^ In a healthy adult, SCs are normally dormant, but in response to injury, they get activated and undergo symmetric division, giving rise to myoblasts, which are subsequently fused to existing muscle fibers. In dystrophic individuals, the activity of SC can be impaired due to various factors such as SC exhaustion and increased fibrosis, which can prevent the passage of activation signals. Additionally, in some MDs, the mutant gene expressed in SC affects SC performance directly. Accessing SCs through a suitable delivery vehicle can be important especially when delivering transiently expressed gene editing agents to ensure that the corrected gene is supplied to the muscle fiber upon muscle regeneration. The connective tissue supporting the skeletal muscles is represented by a complicated extracellular matrix (ECM) composed of collagens, glycoproteins, and peptidoglycans.^21^ The ECM that wraps around each myofibril is called endomysium, while a separate type of ECM (epimysium) envelopes fascicles, and the entire muscle is surrounded by the third type, called perimysium. The ECM plays a role in evenly distributing the contractive force as well as in focal adhesion. The ECM is linked to the muscle cell cytoskeleton through a dystrophin-associated protein complex. This well-studied interaction is known to not only protect the muscle fibers from mechanical damage but also modulate many critical biochemical processes. The ECM is also known to be filled with various proteins responsible for signaling, ECM remodeling, wound healing, and more.^22^^,^^23^ Moreover, the muscle ECM contains a higher percentage of negatively charged proteins: glycosaminoglycans and proteoglycans.^21^ This environment has to be taken into account when designing delivery strategies for the muscle since it is hypothesized that it can affect the delivery of charged carriers, for example, glycosaminoglycans may be displacing nucleic acids from positively charged particles, hindering the delivery.^24^

Unlike the skeletal muscles, which are distributed throughout the body, cardiac muscles are tightly packed in the heart, where they are present only in the middle layer of the cardiac structure, surrounded by the epicardium and endocardium, which are composed of mesothelial and endothelial cells, respectively (Figure 2B). Cardiac muscles are somewhat similar in structure to skeletal muscles as they consist of similar actin-myosin fibers bundled up into myofibrils inside each cardiac muscle cell or cardiomyocyte.^25^ However, unlike the multinucleated skeletal muscles, cardiomyocytes have a single, centrally located nucleus. Individual myocytes are joined into cylindrical branched fibers via intercalated disks. The tight junctions located in the intercalated disks contain gap junctions whose low resistance allows the transfer of ions from one cardiomyocyte to another, facilitating synchronic depolarization.^26^ Compared to skeletal muscles, cardiomyocytes also present larger and fewer t-tubules, which are invaginations of the sarcolemma that play a role in transmitting the action potential. Cardiac muscles present poor regeneration capacity,^27^ showing below 1% of adult cardiac cell turnover per year.^28^ Similar to the non-dividing skeletal muscle fiber, this feature allows non-integrating payloads to avoid dilution through cell division. Various health conditions can require the delivery of therapeutic agents to the heart, including chronic aging-related conditions. Importantly, conditions that involve cardiac muscles may provide the privilege of local direct delivery through invasive surgical approaches or less invasive catheter-based approaches.^8^ However, when discussing MDs, it is important to appreciate the differences in the level of myocardium involvement in the condition.

## Muscle-targeting gene delivery strategies

### Viral vector-based muscular delivery of MD pathogenic genes

#### Adeno-associated viral vectors

Adeno-associated virus (AAV) is a virus incapable of independent replication, shaped as an icosahedron with 26 nm diameter and a linear single-strand DNA genome of 4.7 kb^29^ AAV-delivered payload persists in the cell in an episomal form.

The first attempts at muscle transductions were made in the 1990s with Fischer et al. demonstrating the efficient transduction of a minigene expressing an *E. coli* β-galactosidase under the control of a CMV promoter.^30^ Over the 30 years since, AAVs have been extensively studied for various MDs, with some examples summarized in Table 2. While AAV-9 became the go-to human serotype for MDs due to its very high systemic transduction efficiency and cardiac tropism,^31^ other AAV serotypes such as AAV-1, AAV-6, and AAV-8 were also successfully used for systemic muscle transduction in limb-girdle MD (LGMD),^32^ facioscapulohumeral MD,^33^^,^^34^ and other MDs.^35^^,^^36^^,^^37^ Extensive research has proved that AAVs efficiently transduce muscle fibers, but, as mentioned before, the ability to reach the SC might be an important advantage of a delivery system in MDs. The ability of AAVs to perform this task has been a controversial issue, with some evidence supporting the transduction of SC by AAVs^38^^,^^39^^,^^40^ and others questioning it.^41^ Currently, AAV vectors are by far the most well established for muscular delivery; however, they still suffer from several limitations.

**Table 2:** Summary of advantages and limitations of various delivery strategies

	Advantages	Disadvantages	Examples
**Viral vectors for nucleic acid delivery**			

AAV	cardiac tropism high systemic transduction efficiency some serotypes are capable of transducing SCs with limited efficiency	limited cargo capacity (up to 4.7 kb) pre-existing immunity in up to 60% of patients liver tropism leading to toxicity integration risk	FDA-approved therapy uses rAAVrh74 to deliver a cassette expressing microdystrophin under the control of a muscle-specific MHCK7 promoter^42^ AAV serotypes such as AAV-1, AAV-6, and AAV-8 were used for systemic muscle transduction in LGMD,^32^ FSHD,^33^^,^^34^ and other MDs^35^^,^^36^^,^^37^ dual-AAVs were used to deliver parts of Cas9 that are later joined at the protein level by inteins^43^^,^^44^ AAV was used to deliver minimized base editor nSpCas9-miniABE(GG) for DMD point mutation correction^45^
Lentiviruses (LVs)	long-term expression amendable to pseudotyping rare pre-existing immunity	packaging up to 9 kb unpredictable integration sites innate immune response	integrating LVs successfully induced microdystrophin expression in adult mice^46^^,^^47^ LV pseudotyped with fusogens Myomaker and Myomerger injected locally and systemically to deliver microdystrophin^48^

**Non-viral vectors for nucleic acid delivery**			

Lipid nanoparticles (LNPs)	the possibility of re-administration higher packaging capacity simpler large-scale production	clearance by the liver low muscle transduction	Cas9-mediated exon skipping in DMD after intramuscular injection of LNP^49^ intramuscular delivery of Cas9 ribonucleoprotein^50^ systemic delivery into infarcted myocardium^51^ *in utero* delivery to the heart^52^
Extracellular vesicles (EVs)	the possibility of re-administration higher packaging capacity	challenging manufacturing and mRNA loading	intramuscular injection of red blood cell-derived EVs loaded with myostatin-targeting siRNA^53^^,^^54^ myogenic progenitor cell-derived exosomes administered to DMD-deficient mice administered directly into the myocardium^53^^,^^54^ systemic injection of cardiosphere-derived cells to deliver mRNA intramyocardially and intramuscularly^55^
Polymer nanoparticles (PNPs)	the possibility of re-administration higher packaging capacity simpler large-scale production increased dystrophic muscle tropism	only shown to be applicable to ASO delivery	PEGDB-based particles injected intravenously into DMD model^56^ PEG-fibrinogen microsphere-based delivery of ASO^57^
Virus-like particles (VLPs)	can be pseudotyped to allow for more specific delivery not limited in packaging capacity the delivered nucleic acid remains unintegrated into the host genome	poorly studied in muscles	has not been tested in muscles to date

**Assistive technologies**			

Electro-enhanced plasmid transfer	can be used to deliver naked nucleic acids	low efficiency only applicable to distal muscles	intramuscular injection of plasmid DNA with muscle electroporation^58^
Isolated limb vein injection	can be used in combination with one of the delivery vehicles increases the level of gene delivery to distal muscles limits the toxicity associated with off-target delivery	only applicable to distal muscles high volumes of injection can damage the peripheral nerves^59^ uneven levels of transgene expression in different muscles^60^	clinical trial performed isolated limb infusion (a method which does not require high pressure) with an AAV carrying the *SGCA* gene for the treatment of LGMD2D^61^
Ultrasound-combined nanobubbles and microbubbles	can be used in combination with one of the delivery vehicles may be functionalized by attaching peptides or antibodies^62^ increase the level of gene delivery to distal muscles biodegradable	poorly studied in muscles applicable to tissues that can be treated by ultrasound	intramuscular delivery of plasmid DNA-loaded acoustic liposomes^63^ PEG-based NBs were used to deliver luciferase plasmid DNA intramuscularly and via limb vein perfusion with ultrasound assistance^64^

Selected papers using these approaches are presented as examples.

Unfortunately, the use of AAVs is impaired by their limited cargo capacity (up to 4.7 kb), since many MD-causing genes are much larger (e.g., DMD, 11 kb; DYSF, 6.9 kb). To overcome this limit, the strategy of minigenes was created. A minigene is the result of downsizing a full protein to a minimal functional unit. For example, over 30 microdystrophins were developed with up to 75% reduction of the coding sequence.^10^ Possible downsides of this strategy include reduced functionality and immune reaction to the protein sequence.^10^^,^^65^

In addition, any AAV treatment is currently considered to be a “one-shot therapy” due to the rapid appearance of neutralizing antibodies (nAbs) that prevent repetitive injections. Some patients might even present pre-existing nAbs making them ineligible to receive the treatment. The estimated rate of pre-existing nAbs to wild-type AAV serotypes can be up to 60% in certain populations.^66^

Next, although the wild-type AAVs show a low rate (0.1%) of genomic integration, limited to a safe harbor location on chromosome 19, this is not true for recombinant vectors.^67^^,^^68^ Several studies reported recombinant AAV (rAAV) integration in the Rian locus in the liver, which can be associated with the risk of hepatocellular carcinoma.^69^^,^^70^^,^^71^ More recently, a study in dogs reported 1,741 rAAV integration sites, including 5 genes (EGR2, EGR3, CCND1, LTO1, and ZNF365), associated with transformation in humans. While clonal expansion of cells carrying the integration was reported in this long-term study, no evidence of tumors was found during the period of observation ranging from 2 to 10 years^70^ Moreover, AAV integration has been detected at a high rate when used to deliver Cas9, for example, up to 47% of reads between two double-stranded breaks contained an AAV insertion upon muscle transduction *in vivo*.^72^ The viral genome can be integrated as a full genome, a fragment, or a concatemer.

Finally, the most advanced AAVs for muscle-tropic delivery vehicles pose a significant concern because a high dose (2E+14 vg/kg) is required to reach all of the affected muscles,^10^ and high-dose treatments have been associated with patient deaths in the past.^73^ In 2022, a lethal case occurred, involving a 27-year-old man treated with an AAV-9 delivering a Cas9-based transactivator at 1E+14 vg/kg.^74^ Minimal transgene expression was detected, and no anti-AAV-9 antibodies and T cells were found, so it was hypothesized that innate immune reaction induced the cytokine-mediated capillary leak. Increased amounts of interleukin-6 were seen, especially in the pericardial fluid. Importantly, before the treatment, the patient presented with a significantly decreased muscle mass (45%), a restrictive pulmonary defect, and mild left ventricular systolic dysfunction, possibly impairing his resistance to the acute toxicity of the AAV treatment. A similar case occurred with another, 16-year-old patient treated with 2E+14 vg/kg.^75^ However, patients in previous clinical trials were dosed with comparable doses (2E+14 vg/kg), with no serious adverse effects.^76^ In a different study, where AAVs were evaluated as a potential treatment for myotubule myopathy, 4 deaths occurred: 1 in the lower dose cohort (1.3E+14) and 3 in the higher dose cohort (3.5E+14).^76^ In this case, while all of them presented with cholestatic liver failure at the time of death, the study found that a significant percent of the participants had pre-existing histories of hepatobiliary diseases. In general, 11 AAV-treated patients died in recent years, due to different acute reactions.^73^ Therefore, these cases require thorough investigation and suggest that our understanding of AAV-related toxicity is incomplete.

The first AAV-based muscle-directed therapy for DMD was approved in the summer of 2023 following the NCT05096221 clinical trial.^42^ The approved therapy (delandistrogene moxeparvovec) uses rAAVrh74 to deliver a cassette expressing microdystrophin under the control of a muscle-specific MHCK7 promoter.^76^ The AAVrh74 is a serotype isolated from rhesus monkeys, chosen due to the low abundance of nAbs in the patient population (∼15%), its relatively low immunogenicity, and its high muscle and cardiac tropism.^77^ Although the final results remain to be published, in the pilot study, a robust microdystrophin expression was achieved (81.2% of muscle fibers, 96% at the sarcolemma) with patients reporting mild side effects.^76^ The patients exhibited improved functional test scores and reduced creatine kinase levels for at least a year after injection. However, the improvement varied between patients, which ultimately led the FDA to limit the use of delandistrogene moxeparvovec for patients between 4 and 5 years old, deeming the risk-benefit profile as unfavorable for older ages.^42^ The low efficiency of the therapy in older kids is likely caused by the marked muscle loss that already occurred by this point in life. Importantly, it should be noted that delandistrogene moxeparvovec was never expected to cure DMD but rather to convert it into the milder Becker MD (BMD) due to the drastically shorter amino acid sequence of microdystrophin, which lacks important functional domains.

Naturally, AAV-based strategies for gene editing are widely explored. Unfortunately, the CRISPR-Cas9 system is also too large to fit into a single AAV; creative approaches are explored such as dual-AAVs delivering parts of Cas9 that are later joined at the protein level by inteins^43^^,^^44^ or RNA *trans*-splicing,^78^ as well as minimizing Cas-effectors such as mini base editors (nSpCas9-miniABE(GG)).^45^ A notable attempt was made by Xu et al., who designed a novel variant of base editor with low off-target editing and delivered it *in vivo* in the form of split editors joined by inteins to edit a specific DMD-causing point mutation, achieving almost complete restoration in the heart with a significantly lower dystrophin restoration in gastrocnemius (5% of dystrophin-positive fibers).^45^ This difference is explained by the authors by the selective advantage of transduced cardiomyocytes, as well as the possible spreading of the delivered transcript through cardiomyocyte-derived extracellular vesicles (EVs). Yet again, it is important to note that the gene editing approach has to be tailored to specific mutations. Furthermore, AAVs can also be a less favorable way of delivering gene editing constructs due to their long persistence in the cells, which can lead to increased off-target editing, DNA damage response, and immune reaction.^79^^,^^80^^,^^81^

While there are still obstacles in the way of the wider utilization of AAV-based therapies, considerable efforts are ongoing to overcome them. For example, several research groups focus on altering the AAV capsid to increase the potency and selectivity of muscle transduction using directed evolution,^82^ or semirational bioengineering.^83^ Another strategy is liver de-targeting, which allows for an increased dose to be injected.^84^ There is also a hypothesis that non-natural serotypes would not be subjected to neutralization by pre-existing antibodies. In addition, coating the vector was used to increase muscle tropism^85^ and avoid antibody-based neutralization.^86^ However, there are no approaches capable of increasing AAV packaging capacity. Therefore, while considering the advantages of AAV vectors, there is still a great need for new delivery vehicles supporting both the delivery of large genes and redosing.

#### Lentiviral vectors

Lentiviruses (LVs) are a subtype of retroviruses, enveloped viruses with an RNA genome, which undergoes reverse transcription and integrates into the host genome.^87^ LVs may present some advantages for MD therapy such as long-term persistence even in dividing cells, higher packaging capacity, and rare cases of pre-existing immunity.

Several attempts have been made to use LVs in MD treatments, and while integrating LVs have been able to induce microdystrophin expression in adult mice,^46^^,^^47^ systemic delivery was not possible due to the immune response to LV injection.^88^ As enveloped viruses, LVs are amendable to pseudotyping—adding a protein on the envelope to target specific cells. Previously, LVs pseudotyped with viral fusion proteins were shown to transduce muscle and satellite cells.^89^^,^^90^ A different method of targeting was recently developed by Hindi et al. and uses a genome-integrating LV pseudotyped with fusogens—proteins whose main function is to assist membrane fusion in cases such as the fusion of myoblast with the muscle fiber.^48^ In this case, the fusogens Myomaker and Myomerger are involved. The mice were dosed systemically 3 times reaching up to 80% of positive fibers in the diaphragm and 10% in the limbs; however, the heart was not transduced. The neutralization effect was not observed on the efficiency of the second and third injections.

Unfortunately, LVs suffer from several shortcomings mostly related to their safety profile. LVs were actively explored as a gene delivery tool in the 2000s but lost their momentum in the field of *in vivo* delivery due to safety concerns. Even though their ability to integrate into the genome allows long-term expression, their integration sites are mostly unpredictable except for their preference for highly transcribed genes. Furthermore, highly expressed constructs were even shown to activate neighboring genes^87^; therefore, LVs are valuable as an *ex vivo* delivery tool where their safe integration can be confirmed, but not as an *in vivo* delivery system. Subsequently, integrase-deficient LVs were created to avoid the risk of insertional mutagenesis, with such vectors persisting in the cells as episomes.^47^ Although pre-existing immunity is uncommon, LVs induce an innate immune response, likely due to the DNA intermediate produced from their viral genome during reverse transcription.^91^^,^^92^ Additionally, LVs efficiently transduce antigen-presenting cells, leading to the immune response to the transgene protein itself.^88^ While LVs are capable of packaging up to 9 kb,^93^ which is more than AAVs but still limiting, attempts were made to increase the capacity to deliver a full DMD coding sequence using template switching and heterozygous co-packaging that allows inducing recombination during reverse transcription.^94^ Although a couple of research groups published new approaches to LV vector usage for muscle transduction, it is unlikely that they could be applied to MD therapies until the safety concerns are resolved.^90^

### Non-viral muscular delivery of nucleic acids

#### Lipid nanoparticles

After the unprecedented success of the COVID-19 mRNA-based vaccines, lipid nanoparticles (LNPs) took a central role as a non-viral delivery vehicle for gene therapies. LNPs typically consist of four main components: amino ionizable lipids, phospholipids, cholesterol, and polyethylene glycol (PEG)-functionalized lipids.^95^ LNPs are capable of encapsulating nucleic acids due to the electrostatic interaction of the ionizable lipid’s positive charge with the negatively charged phosphate backbone. Libraries of ionizable lipids are screened to find more stable formulations and to increase intracellular delivery. While ionizable lipids are neutral at physiological pH, they become cationic at lower pH, supporting both mRNA encapsulation during LNP formulation and the escape of the mRNA into the cytoplasm after endocytosis.^96^ Phospholipids are helper lipids required for the LNP structure, while cholesterol is required for particle stability, and PEG leads to increased circulation time and decreased immunogenicity.^97^ By manipulating the type of lipids, the charge, the size, and the composition of the nanoparticle, one can potentially achieve preferred organ targeting. In addition to passive targeting, LNPs can also be functionalized by embedding targeting moieties on their exterior to enable active targeting.

The main advantages of non-viral delivery methods are (1) the possibility of re-administration, (2) higher packaging capacity, and (3) simpler large-scale production.

LNPs were used for gene correction of DMD with limited efficiency. A new ionizable lipid TCL053 was used to formulate LNPs (TCL053/DPPC/cholesterol/DMG-PEG [60:10.6:27.3:2.1]) encapsulating Cas9 mRNA and dual single-guide RNAs (sgRNAs) separately and to induce exon skipping upon intramuscular injection into the gastrocnemius muscle.^49^ The exon skipping achieved about 15% of DMD expression and was stable for over 12 months, in comparison to clinically approved ASOs that only persisted for 1 month. No significant inflammation occurred, so the LNPs were administered up to 3 times with increasing effect. In this study, LNPs were also delivered via intravenous limb perfusion to limit the editing in off-target tissues, such as the liver or germ cells and to treat a larger number of muscle groups. However, it is important to note that in addition to only applying to limb muscles, this method does not prevent liver uptake, since the transgene expression can still be detected in the liver even in a small-volume intramuscular injection.^98^ Additional examples of intramuscular injection in an MD model include Cas9/sgRNA ribonucleoprotein injection resulting in 4.2% dystrophin restoration.^50^ A promising strategy for finding efficient LNP compositions is a high-throughput screening using barcoded mRNA. Using this method, Guimaraes et al. identified several compositions with slightly increased levels of muscle transfection following intravenous administration.^99^ However, as of today, no assessment of therapeutic potential has been done.

More success was achieved when LNPs were used to deliver mRNA into the cardiac muscles of mice, rats, and pigs.^100^ Naked mRNA encoding VEGF was clinically explored as a way to treat myocardial infarction, and the relative success of the strategy prompted researchers to explore LNPs as a delivery vehicle for infarcted myocardium.^101^ When LNPs were delivered systemically, they showed some accumulation in the heart, although mRNA expression was much higher in the infarcted myocardium, compared to a healthy heart.^51^ A special case of LNP delivery to muscles was demonstrated by K. Gao et al.^52^ In this study, LNPs were injected *in utero* at the gestational age of E15.5 and showed 5.5% delivery to the heart 3 h post injection. Around 51%, 37%, and 24% of myofibers in the diaphragm, heart, and skeletal muscle (respectively) expressed tdTomato 4 weeks after birth. Although this method seems to be fairly effective, it raises ethical concerns and as such is not likely to be clinically translated any time soon. Although the mRNA-based vaccine successfully delivered the gene into muscle cells after an intramuscular injection,^49^ this injection route is poorly applicable to most MD therapies due to a large volume of affected muscles spread across the body. Therefore, it is essential to develop an LNP with selective targeting of muscle cells for intravenous administration. This task is challenging primarily because of LNP clearance by the liver, but various technologies are studied to overcome it. So far, the greatest success has been achieved in selective targeting of liver,^95^ spleen, lungs, and cancer cells,^102^ but the possibilities of targeting muscles remain poorly explored. Overall, LNPs present a promising delivery vehicle; however, selective and efficient delivery to muscles has not yet been achieved.

#### EVs

EVs are naturally secreted membrane-bound vesicles ranging from 50 to 500 nm^103^ in diameter that play a role in cellular communications and include various types of vesicles, including exosomes and microvesicles.^104^^,^^105^ Due to their natural ability to deliver molecules to other cells, they are considered a promising delivery system for gene therapy. Previously, EVs were widely explored as a method to deliver small interfering RNA (siRNA), microRNA, proteins, or drugs.^106^ Cell-derived vesicles (CDVs) are produced from cells by extrusion and overcome certain limitations in exosome manufacturing while being similar in size, morphology, and composition.^107^ Due to the natural membrane protein composition, CDVs exhibit tropism to certain tissues, even though the exact mechanism is unclear. Alternatively, targeting moieties can be added to the CDV’s surface by genetic modification of the parent cells or by post-isolation methods.^106^ For example, PROKR1-labeled CDVs have been shown to distribute preferentially to the liver and soleus muscle after intravenous injection.^108^

EVs can be derived from different sources to exhibit different properties. For example, intramuscular injection of red blood cell-derived EVs loaded with myostatin-targeting siRNA was shown to repress myostatin expression in mouse quadriceps, while myogenic progenitor cell-derived exosomes administered to DMD-deficient mice partially restored DMD protein expression in the heart when administered directly into the myocardium.^53^^,^^54^ Aminzadeh et al. used exosomes isolated from cardiosphere-derived cells to deliver mRNA intramyocardially and intramuscularly and also achieved partial transient dystrophin restoration.^55^ An important thing to note is that upon systemic injection, those exosomes were also detected in the brain, liver, lung, spleen, gut, and kidney, indicating high potential for off-target activity. EVs engineered with proteins of viral origin to envelop ribonucleoproteins are promising as a transient gene editor delivery tool. An example of such an approach is termed “NanoMEDIC”; produced in HEK293T cells, these particles employ an FRB dimerization system that facilitates interaction between two fusion proteins upon the addition of rapamycin analog to the producer cells.^109^ Devoid of any targeting mechanism, these particles were capable of editing various cell types (including iPSCs, Jurkat T-lymphocyte cells, and U937 monocyte cells) and demonstrated 7% exon-skipping efficiency when injected into the mouse gastrocnemius.

Despite significant progress, the manufacturing efficiency of EVs is still an important concern. Moreover, payload loading is also technologically challenging and involves post-isolation electroporation or transfection of the parent cells.^105^ Overall, while CDVs or exosomes have the potential to be used as a delivery vehicle in MD therapy, attempts to do so are currently rare and lack a proper assessment of organ specificity.

#### Polymer nanoparticles

Polymer nanoparticles (PNPs) are generally defined as colloidal nanospheres of around 100–500 nm made of various natural or synthetic polymers, including polypeptides, PEG, poly-lactic-acid-co-glycolic-acid, etc.^110^ PNPs have been explored in various indications and have reached clinical approval as delivery systems for small molecules.^111^ The use of PNPs for systemic muscle-targeted delivery of nucleic acids is still ongoing but has the potential to exhibit similar advantages to LNP-based delivery.

Fletcher et al. demonstrated the delivery of Cas9-sgRNA complex using porous silicon nanoparticles (PSiNPs) covered in poly(ethylene glycol)-b-poly(N,N-dimethylaminomethacrylate-stat-*n*-butylmethacry-late) (PEGDB), which can carry various payloads in their honeycomb-shaped structure.^56^ In the DMD mouse model, intramuscular injection led to 100% editing observed at the injection site. Upon intravenous injection into the model of induced muscle inflammation, PSiNPs were found in the tibialis anterior, although in a moderate percentage. A higher level of delivery was detected in the injured leg, compared to the opposite one, indicating that the biodistribution of the particles is affected by the state of the muscle or by vascular leakiness. This phenomenon was also clearly demonstrated by Hicks et al. who showed that mesoporous silica nanoparticles are ten times more likely to be distributed to regenerating muscles, which simultaneously draw them away from the liver and spleen to prevent off-target activity that can lead to toxicity and immune reaction.^112^ Polymers were widely used to enhance the delivery of ASOs,^57^^,^^113^^,^^114^ restoring up to 40% of dystrophin expression by exon skipping. In particular, Cohen et al. proposed that injection of PEG-fibrinogen microspheres into femoral arteries can improve the delivery into limbs and detected nearly 100% of dystrophin-positive fibers, compared to only up to 5% by naked ASOs.^57^ In general, polymeric particles are poorly delivered to the heart, specifically triazine-cored amphiphilic polymer-formulated ASOs induced up to 3% of cardiac muscle fibers to express dystrophin. Yet, it can be considered an improvement compared to naked ASOs, which produced a negligible level of dystrophin in the heart.^57^ Despite some improvements in ASO delivery, it does not necessarily translate into efficient delivery of mRNA or DNA; therefore, studies on mRNA expression after systemic delivery are needed.

#### Virus-like particles

Virus-like particles (VLPs) are an emerging delivery strategy, based on retrovirus-derived polyproteins capable of spontaneous assembly.^115^ Like LVs, VLPs can be pseudotyped to allow for more specific delivery. However, unlike LVs, VLPs are not limited in packaging capacity, and the delivered nucleic acid remains unintegrated into the host genome.

An early attempt to explore VLPs has been made by Prel et al. employing MS2-coat protein interaction with MS2-RNA to construct a chimera by adding them to an HIV-1 vector.^116^ The resulting MS2 chimeric RNA lentiviral particles overcame a restriction of two RNA molecules per particle, caused by the natural mode of RNA packaging in a retrovirus, and contained about 2.8 times more. Upon intramuscular injection, the particles were detected in a 1 cm area of the tight muscle, closely resembling the effect of recombinant LV, which was used in this study as a control. When injected into the caudal vein, the chimeric particle showed no muscle tropism, mainly being detected in the liver and spleen. To our knowledge, VLPs have not been shown to deliver therapeutically relevant RNA to the muscle. In 2021, Banskota et al. presented a new, fourth generation of VLPs, termed engineered VLPs, capable of packaging large amounts of editors in a ribonucleoprotein form.^117^ To our knowledge, they have not yet been tested in muscles; however, they have been shown to deliver prime editing machinery into the mouse cortex with moderate efficiency, achieving 3.2% editing and 15% editing in the retina.^115^ Further research is required to assess the applicability of VLPs to MD therapeutic gene delivery.

### Assistive technologies

#### Electro-enhanced plasmid transfer

Electro-enhanced transfer uses electric pulses to drive the intracellular entry of charged particles, including naked nucleic acids. Using DNA for non-viral gene transfer is still the preferable way to avoid the limitations of viral delivery while preserving the benefits of long-term persistence. In the past, plasmid DNA was found to enter cells poorly on its own. Guha et al. combined intramuscular injection of plasmid DNA with muscle electroporation^58^ and successfully achieved roughly wild-type expression level of the dysferlin transcript (6,952 bp) for up to 3 months. However, it was later shown that dystrophic muscles receive significantly less plasmid upon electroporation, possibly due to muscle fibrosis acting as a physical barrier, highlighting the need to perform delivery studies on corresponding dystrophic animal models.^118^ Notably, lower electroporation levels cannot be explained by fibrosis alone, since the authors did not observe the same effect in older fibrotic mice. Unfortunately, a major limitation of this method is that it is only applicable to distal muscles of limited size.

#### Isolated limb vein injection and hydrodynamic limb injection

Limb perfusion with high volume or high pressure grants obvious advantages over intermuscular injections by increasing the number of muscles transduced and allowing more efficient delivery. It requires the administration of a large volume of solution, while temporarily blocking the blood flow using a tourniquet. It is important to note this method can be applied in combination with one of the previously mentioned delivery vehicles (e.g., LNPs can be administered this way) to avoid clearing by the liver.^49^ Yet, it can only benefit distal muscles and does not apply to proximal muscles such as hips and shoulders, as well as to cardiac and diaphragm muscles that are crucial for DMD treatment. Nonetheless, it can significantly improve the patient’s quality of life when applied to the legs to support walking or to the dominant hand, or for treating MDs that primarily affect distal muscles such as LGMD.

Hydrodynamic limb injection was evaluated in multiple species, including 2 in human studies. One confirmed the safety of high-pressure transvenous limb perfusion in patients with BMD and LGMD by infusing 0.9% saline to their limbs.^59^^,^^119^ Another clinical trial performed isolated limb infusion (a method which does not require high pressure) with an AAV carrying the *SGCA* gene for the treatment of LGMD2D in the lower limbs and showed mild improvement in muscle strength.^61^ To our knowledge, no further clinical trials were initiated based on these studies. The efficiency of hydrodynamic limb injection was mainly evaluated in combination with AAVs,^120^^,^^121^^,^^122^ but some work on plasmid DNA^123^^,^^124^ and LNPs^49^ was also performed. Plasmid DNA delivery yielded a low number of transgene-positive fibers after the first perfusion, but as multiple injections of plasmid DNA are possible, 13% of muscle fibers were dystrophin positive 15 months after the last of 6 injections.^124^

Overall, hydrodynamic limb injection can help to increase the level of gene delivery to distal muscles and limit the toxicity associated with off-target delivery but suffers from major limitations. Not only is it applicable to a small number of affected muscles, but it also often yields uneven levels of transgene expression in different muscles^60^ and, at high volumes of injection, can damage the peripheral nerves.^59^

#### Ultrasound-combined nanobubbles and microbubbles

Nanobubbles (NBs) are shells filled with gas that can be loaded with nucleic acids or drugs. They are capable of extravasation while being stable and fully biodegradable. NBs react to ultrasound by imploding or oscillating, which allows them^125^ to increase membrane permeability and release the NB’s cargo into the cells. NBs can be made of lipids, proteins, or polymers and are sometimes covered in phospholipid-based, nucleic acid-based, or albumin-based coatings to prevent degradation.^62^ In addition, covering the NB with PEG supports the attachment of antibodies or peptides for targeting.

Previously, nanobubbles have been used for gene delivery to tumors and through the blood-brain barrier, and not many studies have been conducted for muscular delivery. One study explored the intramuscular delivery of plasmid DNA-loaded acoustic liposomes (particle range 0.6 nm–7 μm) and achieved luciferase expression for at least 7 days^63^ Recently, PEG-based NBs were used to deliver luciferase plasmid DNA intramuscularly and via limb vein perfusion with ultrasound assistance.^64^ In the latter, the best result was achieved by combining NBs, ultrasound, and a tourniquet with higher ultrasound intensity (>2 W/cm^2^). The delivery of phosphorodiamidate morpholino oligomers (PMOs) for exon skipping was also significantly improved when encapsulated in NBs and assisted by ultrasound. Several similar papers also reported improved PMO delivery with various NB compositions.^126^^,^^127^ At the moment, no intravenous systemic delivery was explored.

**Figure 3:**
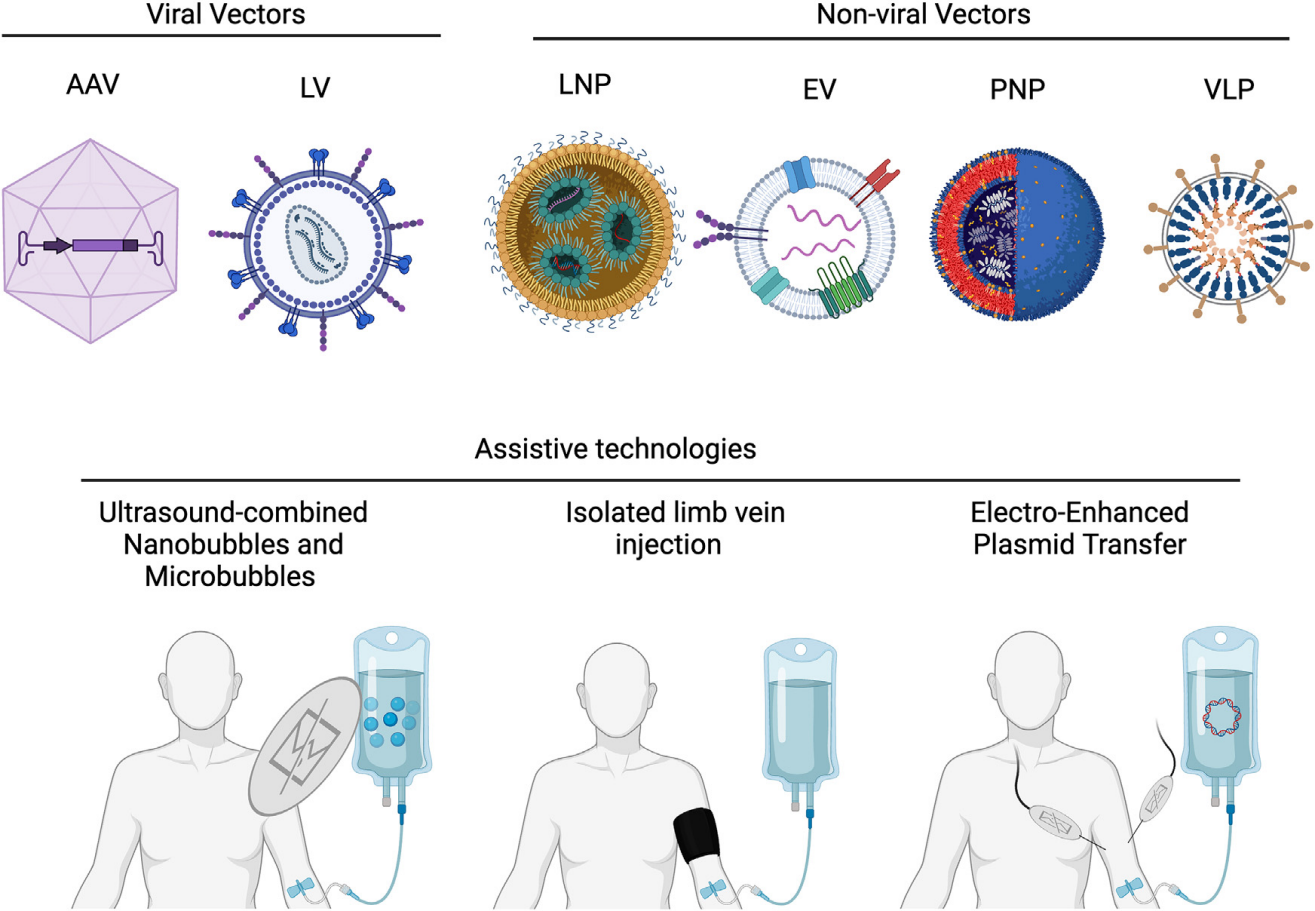
Illustrative summary of muscle-targeting delivery technologies AAV, adeno-associated virus; LV, lentivirus; LNP, lipid nanoparticle; EV, extracellular vesicle; PNP, polymer nanoparticle; VLP, virus-like particle (adapted from An et al.^115^).

The various methods explained in the “Muscle-targeting gene delivery strategies” section are illustrated in Figure 3. The advantages and disadvantages of every method, as well as the papers using each method, can be found summarized in Table 2.

## Future outlook

Currently, there is no effective treatment for any of the MDs. An effective treatment should produce a long-lasting correction of the genetic cause of the disease. Emerging strategies matching these criteria include base and prime editing, DNA “writing”, long-range DNA insertion, and circular RNA with prolonged expression and could be beneficial for patients with MD in general and for patients with DMD in particular.^128^^,^^129^ The applicability of these genetic tools strongly depends on new delivery strategies that would be able to accurately deliver these large constructs to affected muscles.

In summary, MDs constitute a large group of diseases amenable to gene therapy. Over the past decade, significant advances were made in taking gene therapies “from the bench to the bedside.” Nonetheless, currently available treatment options are still limited to a very small percentage of the patient population, and further progress is hindered by the imperfection of the available delivery systems.

AAVs are currently the most advanced delivery tool for MDs. Among the available options, they display the highest transduction efficiency but suffer from limited packaging capacity. AAVs have now reached clinical use as muscle-directed vectors, as well as being used for the treatment of other genetic disorders.

Despite the vaccine-related fame of LNPs, non-viral delivery vehicles are still lacking when it comes to MD therapeutics. Mostly, they suffer from poor muscle transduction efficiency and high liver tropism, making them mostly applicable for intramuscular administration. However, as more research is done on the mechanisms of liver de-targeting, it is possible that they could be used for systemic injections. At the moment, mRNA delivery via non-viral techniques seems to be the most appropriate for the gene editing approach, due to their transient nature.

Finally, several research papers highlighted the difference in particle biodistribution in healthy and dystrophic mice. In this review, we also mentioned the difference in the combination of affected muscles. Therefore, when discussing the biodistribution of a certain system, one has to consider that delivery studies must be performed in a corresponding animal model.

In conclusion, creating a safe, stable, and muscle-specific delivery platform could revolutionize the field of gene therapy and significantly improve the lives of patients with MD. With considerable effort devoted to this area, it is possible that significant improvements in muscle delivery will be seen in the next few years.

## Author contributions

Writing – original draft, Y.C.; writing – review and editing, D.B. and D.P.; supervision, D.P.

## Declaration of interests

D.P. receives licensing fees (to patents on which he was an inventor) from, invested in, consults (or on scientific advisory boards or boards of directors), or is a founder of, or conducts sponsored research at TAU for the following entities: ART Bioscience; BioNTech SE; Earli, Inc.; Kernal Biologics; Geneditor Biologics, Inc.; New Phase Ltd.; NeoVac Ltd.; RiboX Therapeutics; Roche; SirTLab Corporation; and Teva Pharmaceuticals, Inc.

## References

[1] Mercuri E., Bönnemann C.G., Muntoni F. (2019). Muscular dystrophies. Lancet.

[2] Mah J.K., Korngut L., Dykeman J., Day L., Pringsheim T., Jette N. (2014). A systematic review and meta-analysis on the epidemiology of Duchenne and Becker muscular dystrophy. Neuromuscul. Disord..

[3] Taghizadeh E., Rezaee M., Barreto G.E., Sahebkar A. (2019). Prevalence, pathological mechanisms, and genetic basis of limb-girdle muscular dystrophies: A review.. J. Cell. Physiol..

[4] Deenen J.C.W., Arnts H., Van Der Maarel S.M., Padberg G.W., Verschuuren J.J.G.M., Bakker E., Weinreich S.S., Verbeek A.L.M., Van Engelen B.G.M. (2014). Population-based incidence and prevalence of facioscapulohumeral dystrophy. Neurology.

[5] LoRusso S., Weiner B., Arnold W.D. (2018). Myotonic Dystrophies: Targeting Therapies for Multisystem Disease. Neurotherapeutics.

[6] Lawlor M.W., Dowling J.J. (2021). X-linked myotubular myopathy. Neuromuscul. Disord..

[7] Mah J.K., Korngut L., Fiest K.M., Dykeman J., Day L.J., Pringsheim T., Jette N. (2016). A Systematic Review and Meta-analysis on the Epidemiology of the Muscular Dystrophies. Can. J. Neurol. Sci..

[8] Laporte J., Blondeau F., Buj-Bello A., Mandel J.L. (2001). The myotubularin family: From genetic disease to phosphoinositide metabolism. Trends Genet..

[9] Yamashita S. (2021). Recent Progress in Oculopharyngeal Muscular Dystrophy. J. Clin. Med..

[10] Duan D. (2018). Systemic AAV Micro-dystrophin Gene Therapy for Duchenne Muscular Dystrophy. Mol. Ther..

[11] Koenig M., Monaco A.P., Kunkel L.M. (1988). The complete sequence of dystrophin predicts a rod-shaped cytoskeletal protein. Cell.

[12] Nie T., Heo Y.A., Shirley M. (2023). Vutrisiran: A Review in Polyneuropathy of Hereditary Transthyretin-Mediated Amyloidosis. Drugs.

[13] Ventura P., Bonkovsky H.L., Gouya L., Aguilera-Peiró P., Montgomery Bissell D., Stein P.E., Balwani M., Anderson D.K.E., Parker C., Kuter D.J. (2022). Efficacy and safety of givosiran for acute hepatic porphyria: 24-month interim analysis of the randomized phase 3 ENVISION study. Liver Int..

[14] Ray K.K., Wright R.S., Kallend D., Koenig W., Leiter L.A., Raal F.J., Bisch J.A., Richardson T., Jaros M., Wijngaard P.L.J. (2020). Two Phase 3 Trials of Inclisiran in Patients with Elevated LDL Cholesterol. N. Engl. J. Med..

[15] Jinek M., Chylinski K., Fonfara I., Hauer M., Doudna J.A., Charpentier E. (2012). A programmable dual-RNA-guided DNA endonuclease in adaptive bacterial immunity. Science.

[16] Chen G., Wei T., Yang H., Li G., Li H. (2022). CRISPR-Based Therapeutic Gene Editing for Duchenne Muscular Dystrophy: Advances, Challenges and Perspectives. Cells.

[17] Dzierlega K., Yokota T. (2020). Optimization of antisense-mediated exon skipping for Duchenne muscular dystrophy. Gene Ther..

[18] Aartsma-Rus A. (2023). The Future of Exon Skipping for Duchenne Muscular Dystrophy. Hum. Gene Ther..

[19] Amantana A., Iversen P.L. (2005). Pharmacokinetics and biodistribution of phosphorodiamidate morpholino antisense oligomers. Curr. Opin. Pharmacol..

[20] Dumont N.A., Bentzinger C.F., Sincennes M.C., Rudnicki M.A. (2015). Satellite cells and skeletal muscle regeneration. Compr. Physiol..

[21] Gillies A.R., Lieber R.L. (2011). Structure and function of the skeletal muscle extracellular matrix. Muscle Nerve.

[22] Theocharis A.D., Skandalis S.S., Gialeli C., Karamanos N.K. (2016). Extracellular matrix structure. Adv. Drug Deliv. Rev..

[23] Sleboda D.A., Stover K.K., Roberts T.J. (2020). Diversity of extracellular matrix morphology in vertebrate skeletal muscle. J. Morphol..

[24] Ruponen M., Rönkkö S., Honkakoski P., Pelkonen J., Tammi M., Urtti A. (2001). Extracellular Glycosaminoglycans Modify Cellular Trafficking of Lipoplexes and Polyplexes. J. Biol. Chem..

[25] Saxton, A., Tariq, M., and Bordoni, B. (2022). Anatomy, Thorax, Cardiac Muscle. 30570976

[26] Mezzano V., Pellman J., Sheikh F. (2014). Cell junctions in the specialized conduction system of the heart. Cell Commun. Adhes..

[27] Becker C., Hesse M. (2020). Role of Mononuclear Cardiomyocytes in Cardiac Turnover and Regeneration. Curr. Cardiol. Rep..

[28] Bergmann O., Zdunek S., Felker A., Salehpour M., Alkass K., Bernard S., Sjostrom S.L., Szewczykowska M., Jackowska T., Dos Remedios C. (2015). Dynamics of Cell Generation and Turnover in the Human Heart. Cell.

[29] Srivastava A., Lusby E.W., Berns K.I. (1983). Nucleotide sequence and organization of the adeno-associated virus 2 genome. J. Virol..

[30] Fisher K.J., Jooss K., Alston J., Yang Y., Haecker S.E., High K., Pathak R., Raper S.E., Wilson J.M. (1997). Recombinant adeno-associated virus for muscle directed gene therapy. Nat. Med..

[31] Geisler A., Jungmann A., Kurreck J., Poller W., Katus H.A., Vetter R., Fechner H., Müller O.J. (2011). MicroRNA122-regulated transgene expression increases specificity of cardiac gene transfer upon intravenous delivery of AAV9 vectors. Gene Ther..

[32] Fougerousse F., Bartoli M., Poupiot J., Arandel L., Durand M., Guerchet N., Gicquel E., Danos O., Richard I. (2007). Phenotypic correction of α-sarcoglycan deficiency by intra-arterial injection of a muscle-specific serotype 1 rAAV vector. Mol. Ther..

[33] Wallace L.M., Saad N.Y., Pyne N.K., Fowler A.M., Eidahl J.O., Domire J.S., Griffin D.A., Herman A.C., Sahenk Z., Rodino-Klapac L.R., Harper S.Q. (2018). Pre-clinical Safety and Off-Target Studies to Support Translation of AAV-Mediated RNAi Therapy for FSHD. Mol. Ther. Methods Clin. Dev..

[34] Bortolanza S., Nonis A., Sanvito F., MacIotta S., Sitia G., Wei J., Torrente Y., Di Serio C., Chamberlain J.R., Gabellini D. (2011). AAV6-mediated systemic shRNA delivery reverses disease in a mouse model of facioscapulohumeral muscular dystrophy. Mol. Ther..

[35] Şahin İ.O., Özkul Y., Dündar M. (2021). Current and future therapeutic strategies for limb girdle muscular dystrophy type r1: Clinical and experimental approaches. Pathophysiology.

[36] Strings-Ufombah V., Malerba A., Kao S.C., Harbaran S., Roth F., Cappellari O., Lu-Nguyen N., Takahashi K., Mukadam S., Kilfoil G. (2021). BB-301: a silence and replace AAV-based vector for the treatment of oculopharyngeal muscular dystrophy. Mol. Ther. Nucleic Acids.

[37] Marsh S., Hanson B., Wood M.J.A., Varela M.A., Roberts T.C. (2020). Application of CRISPR-Cas9-Mediated Genome Editing for the Treatment of Myotonic Dystrophy Type 1. Mol. Ther..

[38] Kwon J.B., Ettyreddy A.R., Vankara A., Bohning J.D., Devlin G., Hauschka S.D., Asokan A., Gersbach C.A. (2020). In Vivo Gene Editing of Muscle Stem Cells with Adeno-Associated Viral Vectors in a Mouse Model of Duchenne Muscular Dystrophy. Mol. Ther. Methods Clin. Dev..

[39] Tabebordbar M., Zhu K., Cheng J.K.W., Chew W.L., Widrick J.J., Yan W.X., Maesner C., Wu E.Y., Xiao R., Ran F.A. (2016). In vivo gene editing in dystrophic mouse muscle and muscle stem cells. Science.

[40] Nance M.E., Shi R., Hakim C.H., Wasala N.B., Yue Y., Pan X., Zhang T., Robinson C.A., Duan S.X., Yao G. (2019). AAV9 Edits Muscle Stem Cells in Normal and Dystrophic Adult Mice. Mol. Ther..

[41] Arnett A.L., Konieczny P., Ramos J.N., Hall J., Odom G., Yablonka-Reuveni Z., Chamberlain J.R., Chamberlain J.S. (2014). Adeno-associated viral vectors do not efficiently target muscle satellite cells. Mol. Ther. Methods Clin. Dev..

[42] Mullard A. (2023). FDA approves first gene therapy for Duchenne muscular dystrophy, despite internal objections. Nat. Rev. Drug Discov..

[43] Levy J.M., Yeh W.H., Pendse N., Davis J.R., Hennessey E., Butcher R., Koblan L.W., Comander J., Liu Q., Liu D.R. (2020). Cytosine and adenine base editing of the brain, liver, retina, heart and skeletal muscle of mice via adeno-associated viruses. Nat. Biomed. Eng..

[44] Chen Y., Zhi S., Liu W., Wen J., Hu S., Cao T., Sun H., Li Y., Huang L., Liu Y. (2020). Development of Highly Efficient Dual-AAV Split Adenosine Base Editor for In Vivo Gene Therapy. Small Methods.

[45] Xu L., Zhang C., Li H., Wang P., Gao Y., Mokadam N.A., Ma J., Arnold W.D., Han R. (2021). Efficient precise in vivo base editing in adult dystrophic mice. Nat. Commun..

[46] Eren S.A., Tastan C., Karadeniz K.B., Turan R.D., Cakirsoy D., Kancagi D.D., Yilmaz S.U., Oztatlici M., Oztatlici H., Ozer S. (2023). Lentiviral Micro-dystrophin Gene Treatment into Late-stage mdx Mice for Duchenne Muscular Dystrophy Disease. Curr. Gene Ther..

[47] Li S., Kimura E., Fall B.M., Reyes M., Angello J.C., Welikson R., Hauschka S.D., Chamberlain J.S. (2005). Stable transduction of myogenic cells with lentiviral vectors expressing a minidystrophin. Gene Ther..

[48] Hindi S.M., Petrany M.J., Greenfeld E., Focke L.C., Cramer A.A.W., Whitt M.A., Khairallah R.J., Ward C.W., Chamberlain J.S., Podbilewicz B. (2023). Enveloped viruses pseudotyped with mammalian myogenic cell fusogens target skeletal muscle for gene delivery. Cell.

[49] Kenjo E., Hozumi H., Makita Y., Iwabuchi K.A., Fujimoto N., Matsumoto S., Kimura M., Amano Y., Ifuku M., Naoe Y. (2021). Low immunogenicity of LNP allows repeated administrations of CRISPR-Cas9 mRNA into skeletal muscle in mice. Nat. Commun..

[50] Wei T., Cheng Q., Min Y.L., Olson E.N., Siegwart D.J. (2020). Systemic nanoparticle delivery of CRISPR-Cas9 ribonucleoproteins for effective tissue specific genome editing. Nat. Commun..

[51] Evers M.J.W., Du W., Yang Q., Kooijmans S.A.A., Vink A., van Steenbergen M., Vader P., de Jager S.C.A., Fuchs S.A., Mastrobattista E. (2022). Delivery of modified mRNA to damaged myocardium by systemic administration of lipid nanoparticles. J. Control. Release.

[52] Gao K., Li J., Song H., Han H., Wang Y., Yin B., Farmer D.L., Murthy N., Wang A. (2023). In utero delivery of mRNA to the heart, diaphragm and muscle with lipid nanoparticles. Bioact. Mater..

[53] Su X., Jin Y., Shen Y., Ju C., Cai J., Liu Y., Kim I.M., Wang Y., Yu H., Weintraub N.L. (2018). Exosome-Derived Dystrophin from Allograft Myogenic Progenitors Improves Cardiac Function in Duchenne Muscular Dystrophic Mice. J. Cardiovasc. Transl. Res..

[54] Su X., Shen Y., Jin Y., Jiang M., Weintraub N., Tang Y. (2019). Purification and transplantation of myogenic progenitor cell derived exosomes to improve cardiac function in duchenne muscular dystrophic mice. J. Vis. Exp..

[55] Aminzadeh M.A., Rogers R.G., Fournier M., Tobin R.E., Guan X., Childers M.K., Andres A.M., Taylor D.J., Ibrahim A., Ding X. (2018). Exosome-Mediated Benefits of Cell Therapy in Mouse and Human Models of Duchenne Muscular Dystrophy. Stem Cell Rep..

[56] Fletcher R.B., Stokes L.D., Kelly I.B., Henderson K.M., Vallecillo-Viejo I.C., Colazo J.M., Wong B.V., Yu F., d’Arcy R., Struthers M.N. (2023). Nonviral In Vivo Delivery of CRISPR-Cas9 Using Protein-Agnostic, High-Loading Porous Silicon and Polymer Nanoparticles. ACS Nano.

[57] Cohen S.A., Bar-Am O., Fuoco C., Saar G., Gargioli C., Seliktar D. (2022). In vivo restoration of dystrophin expression in mdx mice using intra-muscular and intra-arterial injections of hydrogel microsphere carriers of exon skipping antisense oligonucleotides. Cell Death Dis..

[58] Guha T.K., Pichavant C., Calos M.P. (2019). Plasmid-Mediated Gene Therapy in Mouse Models of Limb Girdle Muscular Dystrophy. Mol. Ther. Methods Clin. Dev..

[59] Fan Z., Kocis K., Valley R., Howard J.F., Chopra M., Chen Y., An H., Lin W., Muenzer J., Powers W. (2015). High-Pressure Transvenous Perfusion of the Upper Extremity in Human Muscular Dystrophy: A Safety Study with 0.9% Saline. Hum. Gene Ther..

[60] Vigen K.K., Hegge J.O., Zhang G., Mukherjee R., Braun S., Grist T.M., Wolff J.A. (2007). Magnetic resonance imaging-monitored plasmid DNA delivery in primate limb muscle. Hum. Gene Ther..

[61] Mendell J.R., Chicoine L.G., Al-Zaidy S.A., Sahenk Z., Lehman K., Lowes L., Miller N., Alfano L., Galliers B., Lewis S. (2019). Gene Delivery for Limb-Girdle Muscular Dystrophy Type 2D by Isolated Limb Infusion. Hum. Gene Ther..

[62] Endo-Takahashi Y., Negishi Y. (2020). Microbubbles and nanobubbles with ultrasound for systemic gene delivery. Pharmaceutics.

[63] Watanabe Y., Horie S., Funaki Y., Kikuchi Y., Yamazaki H., Ishii K., Mori S., Vassaux G., Kodama T. (2010). Delivery of Na/I symporter gene into skeletal muscle using nanobubbles and ultrasound: Visualization of gene expression by PET. J. Nucl. Med..

[64] Sekine S., Mayama S., Nishijima N., Kojima T., Endo-Takahashi Y., Ishii Y., Shiono H., Akiyama S., Sakurai A., Sashida S. (2023). Development of a Gene and Nucleic Acid Delivery System for Skeletal Muscle Administration via Limb Perfusion Using Nanobubbles and Ultrasound. Pharmaceutics.

[65] (2010).

[66] Khatri A., Shelke R., Guan S., Somanathan S. (2022). Higher Seroprevalence of Anti-Adeno-Associated Viral Vector Neutralizing Antibodies Among Racial Minorities in the United States. Hum. Gene Ther..

[67] Deyle D.R., Russell D.W. (2009). Adeno-associated virus vector integration Current Opinion in Molecular Therapeutics. Current Opin. Mole. Therapeu..

[68] Wang Z., Lisowski L., Finegold M.J., Nakai H., Kay M.A., Grompe M. (2012). AAV vectors containing rDNA homology display increased chromosomal integration and transgene persistence. Mol. Ther..

[69] Li Y., Miller C.A., Shea L.K., Jiang X., Guzman M.A., Chandler R.J., Ramakrishnan S.M., Smith S.N., Venditti C.P., Vogler C.A. (2021). Enhanced Efficacy and Increased Long-Term Toxicity of CNS-Directed, AAV-Based Combination Therapy for Krabbe Disease. Mol. Ther..

[70] Nguyen G.N., Everett J.K., Kafle S., Roche A.M., Raymond H.E., Leiby J., Wood C., Assenmacher C.A., Merricks E.P., Long C.T. (2021). A long-term study of AAV gene therapy in dogs with hemophilia A identifies clonal expansions of transduced liver cells. Nat. Biotechnol..

[71] Donsante A., Vogler C., Muzyczka N., Crawford J.M., Barker J., Flotte T., Campbell-Thompson M., Daly T., Sands M.S. (2001). Observed incidence of tumorigenesis in long-term rodent studies of rAAV vectors. Gene Ther..

[72] Hanlon K.S., Kleinstiver B.P., Garcia S.P., Zaborowski M.P., Volak A., Spirig S.E., Muller A., Sousa A.A., Tsai S.Q., Bengtsson N.E. (2019). High levels of AAV vector integration into CRISPR-induced DNA breaks. Nat. Commun..

[73] Duan D. (2023). Lethal immunotoxicity in high-dose systemic AAV therapy. Mol. Ther..

[74] Lek A., Wong B., Keeler A., Blackwood M., Ma K., Huang S., Sylvia K., Batista A.R., Artinian R., Kokoski D. (2023). Death after High-Dose rAAV9 Gene Therapy in a Patient with Duchenne’s Muscular Dystrophy. N. Engl. J. Med..

[75] Lek A., Atas E., Hesterlee S.E., Byrne B.J., Bönnemann C.G. (2023). Meeting Report: 2022 Muscular Dystrophy Association Summit on “Safety and Challenges in Gene Transfer Therapy.”. J. Neuromuscul. Dis..

[76] Mendell J.R., Sahenk Z., Lehman K., Nease C., Lowes L.P., Miller N.F., Iammarino M.A., Alfano L.N., Nicholl A., Al-Zaidy S. (2020). Assessment of Systemic Delivery of rAAVrh74.MHCK7.micro-dystrophin in Children with Duchenne Muscular Dystrophy: A Nonrandomized Controlled Trial. JAMA Neurol..

[77] Goedeker N.L., Dharia S.D., Griffin D.A., Coy J., Truesdale T., Parikh R., Whitehouse K., Santra S., Asher D.R., Zaidman C.M. (2023). Evaluation of rAAVrh74 gene therapy vector seroprevalence by measurement of total binding antibodies in patients with Duchenne muscular dystrophy. Ther. Adv. Neurol. Disord..

[78] Ryu S.M., Koo T., Kim K., Lim K., Baek G., Kim S.T., Kim H.S., Kim D.E., Lee H., Chung E., Kim J.S. (2018). Adenine base editing in mouse embryos and an adult mouse model of Duchenne muscular dystrophy. Nat. Biotechnol..

[79] Liang X., Potter J., Kumar S., Zou Y., Quintanilla R., Sridharan M., Carte J., Chen W., Roark N., Ranganathan S. (2015). Rapid and highly efficient mammalian cell engineering via Cas9 protein transfection. J. Biotechnol..

[80] Pattanayak V., Lin S., Guilinger J.P., Ma E., Doudna J.A., Liu D.R. (2013). High-throughput profiling of off-target DNA cleavage reveals RNA-programmed Cas9 nuclease specificity. Nat. Biotechnol..

[81] Cho S.W., Kim S., Kim Y., Kweon J., Kim H.S., Bae S., Kim J.S. (2014). Analysis of off-target effects of CRISPR/Cas-derived RNA-guided endonucleases and nickases. Genome Res..

[82] Tabebordbar M., Lagerborg K.A., Stanton A., King E.M., Ye S., Tellez L., Krunnfusz A., Tavakoli S., Widrick J.J., Messemer K.A. (2021). Directed evolution of a family of AAV capsid variants enabling potent muscle-directed gene delivery across species. Cell.

[83] El Andari J., Renaud-Gabardos E., Tulalamba W., Weinmann J., Mangin L., Pham Q.H., Hille S., Bennett A., Attebi E., Bourges E. (2022). Semirational bioengineering of AAV vectors with increased potency and specificity for systemic gene therapy of muscle disorders. Sci. Adv..

[84] Zinn E., Unzu C., Schmit P.F., Turunen H.T., Zabaleta N., Sanmiguel J., Fieldsend A., Bhatt U., Diop C., Merkel E. (2022). Ancestral library identifies conserved reprogrammable liver motif on AAV capsid. Cell Rep. Med..

[85] Moretti A., Fonteyne L., Giesert F., Hoppmann P., Meier A.B., Bozoglu T., Baehr A., Schneider C.M., Sinnecker D., Klett K. (2020). Somatic gene editing ameliorates skeletal and cardiac muscle failure in pig and human models of Duchenne muscular dystrophy. Nat. Med..

[86] Matsuzaka Y., Hirai Y., Hashido K., Okada T. (2022). Therapeutic Application of Extracellular Vesicles-Capsulated Adeno-Associated Virus Vector via nSMase2/Smpd3, Satellite, and Immune Cells in Duchenne Muscular Dystrophy. Int. J. Mol. Sci..

[87] Milone M.C., O’Doherty U. (2018). Clinical use of lentiviral vectors. Leukemia.

[88] Shirley J.L., de Jong Y.P., Terhorst C., Herzog R.W. (2020). Immune Responses to Viral Gene Therapy Vectors. Mol. Ther..

[89] Kimura E., Li S., Gregorevic P., Fall B.M., Chamberlain J.S. (2010). Dystrophin delivery to muscles of mdx mice using lentiviral vectors leads to myogenic progenitor targeting and stable gene expression. Mol. Ther..

[90] MacKenzie T.C., Kobinger G.P., Louboutin J.P., Radu A., Javazon E.H., Sena-Esteves M., Wilson J.M., Flake A.W. (2005). Transduction of satellite cells after prenatal intramuscular administration of lentiviral vectors. J. Gene Med..

[91] Liu Y., Li Y., Xue L., Xiao J., Li P., Xue W., Li C., Guo H., Chen Y. (2022). The effect of the cyclic GMP-AMP synthase-stimulator of interferon genes signaling pathway on organ inflammatory injury and fibrosis. Front. Pharmacol..

[92] Lahaye X., Satoh T., Gentili M., Cerboni S., Conrad C., Hurbain I., ElMarjou A., Lacabaratz C., Lelièvre J.D., Manel N. (2013). The Capsids of HIV-1 and HIV-2 Determine Immune Detection of the Viral cDNA by the Innate Sensor cGAS in Dendritic Cells. Immunity.

[93] Naldini L., Blömer U., Gallay P., Ory D., Mulligan R., Gage F.H., Verma I.M., Trono D. (1996). In vivo gene delivery and stable transduction of nondividing cells by a lentiviral vector. Science.

[94] Counsell J.R., Asgarian Z., Meng J., Ferrer V., Vink C.A., Howe S.J., Waddington S.N., Thrasher A.J., Muntoni F., Morgan J.E., Danos O. (2017). Lentiviral vectors can be used for full-length dystrophin gene therapy. Sci. Rep..

[95] Hou X., Zaks T., Langer R., Dong Y. (2021). Lipid nanoparticles for mRNA delivery. Nat. Rev. Mater..

[96] Veiga N., Diesendruck Y., Peer D. (2023). Targeted nanomedicine: Lessons learned and future directions. J. Control. Release.

[97] Godbout K., Tremblay J.P. (2022). Delivery of RNAs to Specific Organs by Lipid Nanoparticles for Gene Therapy. Pharmaceutics.

[98] Du X., Yada E., Terai Y., Takahashi T., Nakanishi H., Tanaka H., Akita H., Itaka K. (2023). Comprehensive Evaluation of Lipid Nanoparticles and Polyplex Nanomicelles for Muscle-Targeted mRNA Delivery. Pharmaceutics.

[99] Guimaraes P.P.G., Zhang R., Spektor R., Tan M., Chung A., Billingsley M.M., El-Mayta R., Riley R.S., Wang L., Wilson J.M., Mitchell M.J. (2019). Ionizable lipid nanoparticles encapsulating barcoded mRNA for accelerated in vivo delivery screening HHS Public Access. J. Control. Release.

[100] Turnbull I.C., Eltoukhy A.A., Fish K.M., Nonnenmacher M., Ishikawa K., Chen J., Hajjar R.J., Anderson D.G., Costa K.D. (2016). Myocardial delivery of lipidoid nanoparticle carrying modRNA induces rapid and transient expression. Mol. Ther..

[101] Anttila V., Saraste A., Knuuti J., Hedman M., Jaakkola P., Laugwitz K.L., Krane M., Jeppsson A., Sillanmäki S., Rosenmeier J. (2023). Direct intramyocardial injection of VEGF mRNA in patients undergoing coronary artery bypass grafting. Mol. Ther..

[102] Kon E., Ad-El N., Hazan-Halevy I., Stotsky-Oterin L., Peer D. (2023). Targeting cancer with mRNA–lipid nanoparticles: key considerations and future prospects. Nat. Rev. Clin. Oncol..

[103] Wang W., Li M., Chen Z., Xu L., Chang M., Wang K., Deng C., Gu Y., Zhou S., Shen Y. (2022). Biogenesis and function of extracellular vesicles in pathophysiological processes of skeletal muscle atrophy. Biochem. Pharmacol..

[104] Sheta M., Taha E.A., Lu Y., Eguchi T. (2023). Extracellular Vesicles: New Classification and Tumor Immunosuppression. Biology.

[105] Cecchin R., Troyer Z., Witwer K., Morris K.V. (2023). Extracellular vesicles: The next generation in gene therapy delivery. Mol. Ther..

[106] Jafari D., Shajari S., Jafari R., Mardi N., Gomari H., Ganji F., Forouzandeh Moghadam M., Samadikuchaksaraei A. (2020). Designer Exosomes: A New Platform for Biotechnology Therapeutics. BioDrugs.

[107] Lunavat T.R., Jang S.C., Nilsson L., Park H.T., Repiska G., Lässer C., Nilsson J.A., Gho Y.S., Lötvall J. (2016). RNAi delivery by exosome-mimetic nanovesicles – Implications for targeting c-Myc in cancer. Biomaterials.

[108] Zhang C., Mok J., Seong Y., Lau H.C., Kim D., Yoon J., Oh S.W., Park T.S., Park J. (2021). PROKR1 delivery by cell-derived vesicles restores the myogenic potential of Prokr1-deficient C2C12 myoblasts. Nanomedicine..

[109] Gee P., Lung M.S.Y., Okuzaki Y., Sasakawa N., Iguchi T., Makita Y., Hozumi H., Miura Y., Yang L.F., Iwasaki M. (2020). Extracellular nanovesicles for packaging of CRISPR-Cas9 protein and sgRNA to induce therapeutic exon skipping. Nat. Commun..

[110] Quintanar-Guerrero D., Allémann E., Fessi H., Doelker E. (1998). Preparation techniques and mechanisms of formation of biodegradable nanoparticles from preformed polymers. Drug Dev. Ind. Pharm..

[111] Malek R., Wu S.T., Serrano D., Tho T., Umbas R., Teoh J., Lojanapiwat B., Ong T.A., On W.K., Thai S.M. (2022). ELIGANT: a Phase 4, interventional, safety study of leuprorelin acetate (ELIGARD®) in Asian men with prostate cancer. Transl. Androl. Urol..

[112] Hicks M.R., Liu X., Young C.S., Saleh K., Ji Y., Jiang J., Emami M.R., Mokhonova E., Spencer M.J., Meng H., Pyle A.D. (2023). Nanoparticles systemically biodistribute to regenerating skeletal muscle in DMD. J. Nanobiotechnology.

[113] Wang M., Wu B., Tucker J.D., Shah S.N., Lu P., Bollinger L.E., Lu Q. (2017). Tween 85-Modified Low Molecular Weight PEI Enhances Exon-Skipping of Antisense Morpholino Oligomer In Vitro and in mdx Mice. Mol. Ther. Nucleic Acids.

[114] Wang M., Wu B., Tucker J.D., Shah S.N., Lu P., Lu Q. (2020). Triazine-cored polymeric vectors for antisense oligonucleotide delivery in vitro and in vivo. J. Nanobiotechnology.

[115] An M., Raguram A., Du S.W., Banskota S., Davis J.R., Newby G.A., Chen P.Z., Palczewski K., Liu D.R. (2024). Engineered virus-like particles for transient delivery of prime editor ribonucleoprotein complexes in vivo. Nat. Biotechnol..

[116] Prel A., Caval V., Gayon R., Ravassard P., Duthoit C., Payen E., Maouche-Chretien L., Creneguy A., Nguyen T.H., Martin N. (2015). Highly efficient in vitro and in vivo delivery of functional RNAs using new versatile MS2-chimeric retrovirus-like particles. Mol. Ther. Methods Clin. Dev..

[117] Banskota S., Raguram A., Suh S., Du S.W., Davis J.R., Choi E.H., Wang X., Nielsen S.C., Newby G.A., Randolph P.B. (2022). Engineered virus-like particles for efficient in vivo delivery of therapeutic proteins. Cell.

[118] Florio F., Accordini S., Libergoli M., Biressi S. (2022). Targeting Muscle-Resident Single Cells Through in vivo Electro-Enhanced Plasmid Transfer in Healthy and Compromised Skeletal Muscle. Front. Physiol..

[119] Fan Z., Kocis K., Valley R., Howard J.F., Chopra M., An H., Lin W., Muenzer J., Powers W. (2012). Safety and feasibility of high-pressure transvenous limb perfusion with 0.9% saline in human muscular dystrophy. Mol. Ther..

[120] Le Guiner C., Servais L., Montus M., Larcher T., Fraysse B., Moullec S., Allais M., François V., Dutilleul M., Malerba A. (2017). Long-term microdystrophin gene therapy is effective in a canine model of Duchenne muscular dystrophy. Nat. Commun..

[121] Le Guiner C., Montus M., Servais L., Cherel Y., Francois V., Thibaud J.L., Wary C., Matot B., Larcher T., Guigand L. (2014). Forelimb Treatment in a Large Cohort of Dystrophic Dogs Supports Delivery of a Recombinant AAV for Exon Skipping in Duchenne Patients. Mol. Ther..

[122] Qiao C., Li J., Zheng H., Bogan J., Li J., Yuan Z., Zhang C., Bogan D., Kornegay J., Xiao X. (2009). Hydrodynamic limb vein injection of adeno-associated virus serotype 8 vector carrying canine myostatin propeptide gene into normal dogs enhances muscle growth. Hum. Gene Ther..

[123] Wooddell C.I., Hegge J.O., Zhang G., Sebestyén M.G., Noble M., Griffin J.B., Pfannes L.V., Herweijer H., Hagstrom J.E., Braun S. (2011). Dose response in rodents and nonhuman primates after hydrodynamic limb vein delivery of naked plasmid DNA. Hum. Gene Ther..

[124] Zhang G., Wooddell C.I., Hegge J.O., Griffin J.B., Huss T., Braun S., Wolff J.A. (2010). Functional efficacy of dystrophin expression from plasmids delivered to mdx mice by hydrodynamic limb vein injection. Hum. Gene Ther..

[125] Su C., Ren X., Nie F., Li T., Lv W., Li H., Zhang Y. (2021). Current advances in ultrasound-combined nanobubbles for cancer-targeted therapy: a review of the current status and future perspectives. RSC Adv..

[126] Negishi Y., Ishii Y., Nirasawa K., Sasaki E., Endo-Takahashi Y., Suzuki R., Maruyama K. (2018). PMO delivery system using bubble liposomes and ultrasound exposure for Duchenne muscular dystrophy treatment. Method. Mole. Biol..

[127] Negishi Y., Ishii Y., Shiono H., Akiyama S., Sekine S., Kojima T., Mayama S., Kikuchi T., Hamano N., Endo-Takahashi Y. (2014). Bubble liposomes and ultrasound exposure improve localized morpholino oligomer delivery into the skeletal muscles of dystrophic mdx mice. Mol. Pharm..

[128] Jiang T., Zhang X.O., Weng Z., Xue W. (2022). Deletion and replacement of long genomic sequences using prime editing. Nat. Biotechnol..

[129] Ferreira da Silva J., Tou C.J., King E.M., Eller M.L., Ma L., Rufino-Ramos D., Kleinstiver B.P. (2023). Click editing enables programmable genome writing using DNA polymerases and HUH endonucleases. bioRxiv.

